# Hepatocyte Transplantation: Cell Sheet Technology for Liver Cell Transplantation

**DOI:** 10.1007/s40472-017-0156-7

**Published:** 2017-08-08

**Authors:** Kohei Tatsumi, Teruo Okano

**Affiliations:** 1 Department of Physiology and Regenerative Medicine, Kindai University Faculty of Medicine, 377-2 Ohno-higashi, Osaka-sayama, Osaka 589-8511 Japan; 2 Institute of Advanced Biomedical Engineering and Science, Tokyo Women’s Medical University, 8-1 Kawada-cho, Shinjuku-ku, Tokyo, 162-8666 Japan; 3 Cell Sheet Tissue Engineering Center and Department of Pharmaceutics and Pharmaceutical Chemistry, University of Utah, Salt Lake City, UT 84112 USA

**Keywords:** Hepatocyte, Transplantation, Cell sheet, Liver disease, Hemophilia

## Abstract

**Purpose of Review:**

We will review the recent developments of cell sheet technology as a feasible tissue engineering approach. Specifically, we will focus on the technological advancement for engineering functional liver tissue using cell sheet technology, and the associated therapeutic effect of cell sheets for liver diseases, highlighting hemophilia.

**Recent Findings:**

Cell-based therapies using hepatocytes have recently been explored as a new therapeutic modality for patients with many forms of liver disease. We have developed a cell sheet technology, which allows cells to be harvested in a monolithic layer format. We have succeeded in fabricating functional liver tissues in mice by stacking the cell sheets composed of primary hepatocytes. As a curative measure for hemophilia, we have also succeeded in treating hemophilia mice by transplanting of cells sheets composed of genetically modified autologous cells.

**Summary:**

Tissue engineering using cell sheet technology provides the opportunity to create new therapeutic options for patients with various types of liver diseases.

## Introduction

The liver is the largest and highest functional complex organ, playing many vital functions including cholanopoiesis, metabolism, detoxification, and protein synthesis. An ultimate treatment for severe liver failure is orthotropic liver transplantation (OLT), but the establishment of OLT as a common therapy is severely hampered by a worldwide shortage of donor livers [1, 2]. As an alternative to OLT, a cell-based therapeutic using hepatocytes, known as hepatocyte transplantation, has been investigated and clinically conducted for liver failure and liver-based metabolic disorders [3–5]. In most cases of clinical hepatocyte transplantation, the hepatocytes which are freshly isolated or frozen dispersed are injected into the spleen or into the portal vein [6, 7]. Although the efficacy and safety of hepatocyte transplantation has been demonstrated in patients with liver-based metabolic disorders such as ornithine transcarbamylase deficiency [8], Crigler–Najjar syndrome [9], glycogen storage disease type I [10], and coagulation factor VII deficiency [3], the therapeutic effects were only partial or transient, and most of the patients needed OLT. The most probable causes of the insufficiency in the effects would be the limitation of number of hepatocytes transplantable into the portal vein without adverse embolism, and the poor engraftment rate of the transplanted hepatocytes in the recipient liver parenchyma, probably elicited by immune and coagulation reaction called instant blood-mediated immune reaction (IBMIR) [11, 12]. It has been assumed that only 2–10% of the number of hepatocytes of the liver mass can be injected at one time for preventing portal hypertension, thrombosis, and microinfarctions [13, 14], and more than 70% of hepatocytes injected into the portal vein are rapidly rejected by IBMIR [15].

Under these current circumstances, an innovative methodological improvement that can achieve more effective cell engraftment and longer-term therapeutic effects is required for the progress of cell-based therapy for liver diseases. Liver tissue engineering, in which the liver cells are engineered and the functional liver tissues are created in vitro and in vivo, would be a promising approach for the purpose [16•]. If the engineered hepatic constructs are engrafted and can maintain their hepatic function at the transplanted sites, this would enable to support the liver function in vivo for a long time period. More recently, we have developed a novel technology of tissue engineering, called cell sheet technology, by which cultured cells can be harvested in a monolayer format (cell sheet) [17–20]. By applying this technology, we have succeeded in fabricating functional liver tissues in subcutaneous spaces of mice by transplanting stacked cell sheets composed of primary hepatocytes [21].

Hemophilia, the most common and severe inherited bleeding disorders, is caused by a lack in the production of functional coagulation factor VIII (FVIII, hemophilia A) or IX (FIX, hemophilia B) [22], resulting in recurrent and spontaneous bleeding in joints and muscles, and sometimes in vital organs. The only treatment currently available is replacement therapy with FVIII or FIX concentrates. This treatment is expensive and not curative because of the requirement of life-long and frequent intravenous infusion of the concentrates. It is known that both FVIII and FIX are produced in the liver [23–26], and that with only a 1–5% of increase in plasma activity levels of the lacked factor, the bleeding symptom of hemophilia patients can be dramatically improved [27]. Therefore, cell-based therapy using liver cells is a feasible, next-generation therapy for hemophilia.

In this paper, we review the current progress of cell-based therapy and tissue engineering approaches including cell sheet technology for liver diseases, especially for hemophilia.

## Cell Sheet Technology

The field of regenerative medicine is defined as a translational research in tissue engineering and molecular biology. This would make it possible to restore damaged tissues and organs to functional ones by stimulating the body’s own repair mechanisms or by transplanting or engineering cells/tissues. Therefore, regenerative medicine is anticipated to be an alternative approach to organ transplantation and is expected to cure patients with diseases that are difficult to treat. It is crucial to develop a technology that enables transplanted cells or engineered tissues to be functionally engrafted for a long time period to maximize the therapeutic effects. As a classical type of cell-based regenerative therapy, the injection of single cell suspension has been widely applied for the treatment of various diseases in clinical settings, owing to their convenience in preparing and handling the cells [28–30]. In this type of cell therapy, however, the cells are normally prepared by enzymatic treatments, leading to cell damages [29]. As a result, only several percent of the prepared cells can be functionally engrafted to target tissues, and this significantly reduces the expected therapeutic effects.

Tissue engineering has been proposed as a promising strategy to overcome these problems [31]. The tissue engineering approach has been developed around the usage of biodegradable materials, such as polyglycolic acid [32], collagen gel [33], fibrin gel [34], and gelatin [35], as a scaffold for the cells. These biodegradable porous polymer scaffolds seeded with cultured cells have been used to fabricate three-dimensional (3D) tissue-like structures. More recently, decellularized scaffolds, which can be used as a naturally occurring 3D biological scaffold for cell culture, has been utilized for constructing more functional whole-organ structures [36]. Although the tissue engineering approach using scaffolds seems to be feasible [37, 38], several barriers still remain, such as fibrosis due to the low cell density in the constructed tissue, necrosis due to the lack of microcirculation, and adverse inflammatory responses leading to the biodegradation of the scaffolds.

**Fig. 1:**
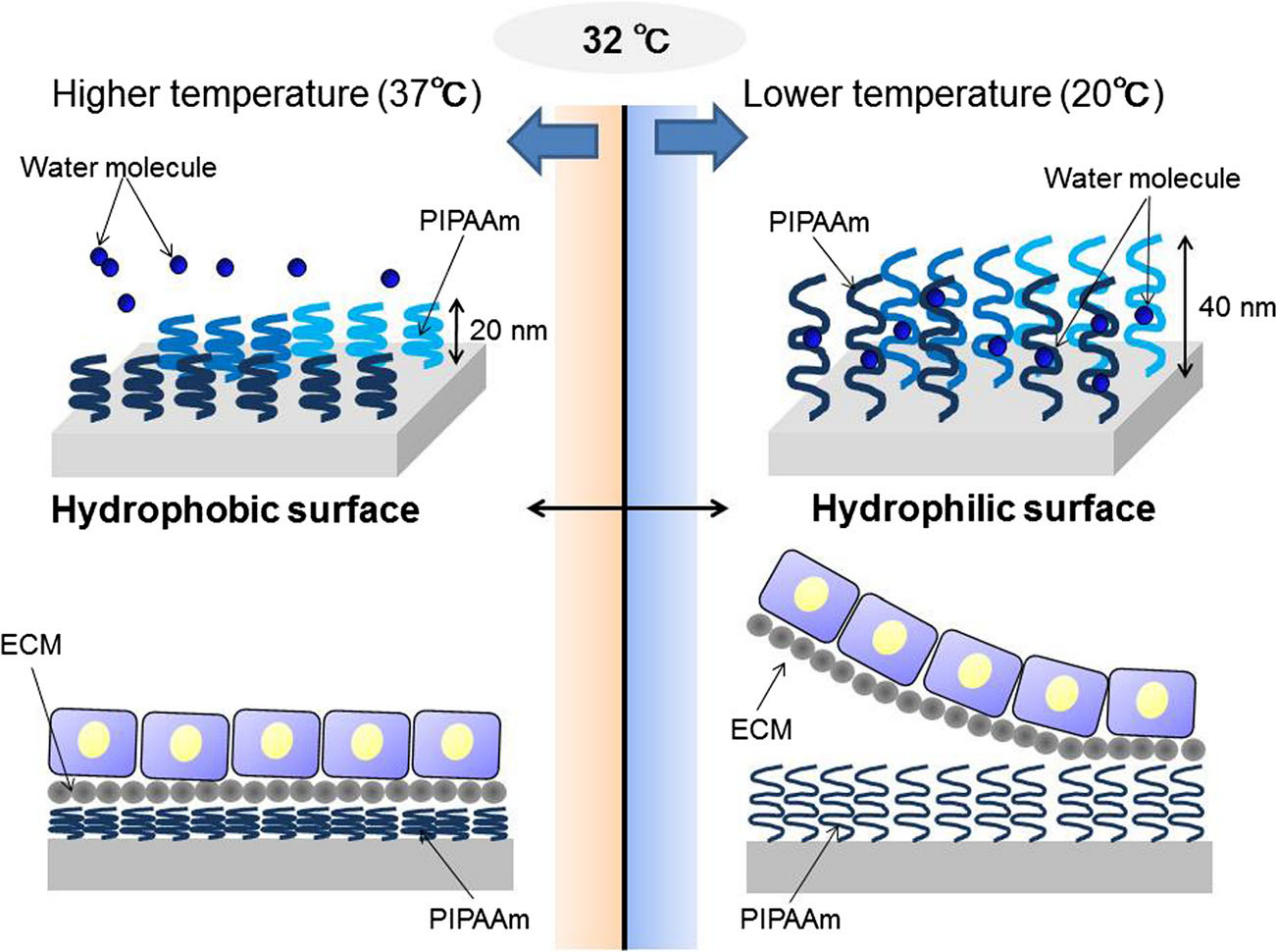
Schematic illustrations of cell sheet engineering using temperature-responsive cell culture dishes. Temperature-responsive polymer poly(N-isopropylacrylamide) (PIPAAm) is covalently grafted on the cell culture plastic dishes at nanometer thickness. This PIPAAm coating provides a slightly hydrophobic surface at the regular culture temperature (37 °C), allowing conventional cell culturing. The lower critical solution temperature of PIPAAm is 32 °C, indicating PIPAAm shows hydrophobic characteristics over 32 °C, whereas it changes to hydrophilic state below 32 °C. Therefore, the PIPAAm coating allows conventional cell culturing at the regular culture temperature (37 °C), but the cultured cells cannot adhere to the surface below 32 °C because of rapid hydration and swelling of the grafted PIPAAm. This results in the spontaneous detachment of the cultured cells from the dishes as a viable monolayer cell sheet format. The PIPAAm remains on the dish surface during the cell detachment process, and the cell sheet maintains the extracellular matrix (ECM) including adhesive protein on their basal side

To resolve these problems, our group has developed an innovative scaffold-free tissue engineering technology, termed “cell sheet-based tissue engineering” [21, 39–52]. The principle of cell sheet engineering is as follows (Fig. 1): cells are cultured on a temperature-responsive cell culture surface. Then, by lowering the culture temperature, the cultured cells are detached from the surface and harvested as an intact monolayer called a “cell sheet,” which can be directly applied for therapeutic use. In the temperature-responsive culture dishes, temperature-responsive polymer poly(N-isopropylacrylamide) (PIPAAm) is covalently grafted on the surface of nanometer thickness [39]. The lower critical solution temperature of PIPAAm is 32 °C. This means PIPAAm shows hydrophobic characteristics over 32 °C, whereas it changes to hydrophilic state below 32 °C. Therefore, the PIPAAm coating allows conventional cell culturing at the regular culture temperature (37 °C), but the cultured cells cannot adhere to the surface below 32 °C because of rapid hydration and swelling of the grafted PIPAAm. This results in the natural detachment of the cells from the surface of culture dishes as a viable monolayer cell sheet format within 1 h. This technology needs no enzymatic digestion to harvest the cells, enabling us to prepare transplantable tissue constructs preserving intact cell–cell contacts and extracellular matrices. Of note, the PIPAAm remains on the culture dish surface during the cell detachment process, meaning the harvested cell sheets contain no PIPAAm components. Furthermore, the cell sheets, which maintain adhesive protein on their basal side, can be transplanted and engrafted to the host tissue without any suturing procedures.

This cell sheet engineering has been applied for fabricating various types of tissue constructs, and several clinical trials have been practically performed for treatments of eye, esophageal, heart, periodontal, and middle ear diseases [53, 54]. Patients with severe corneal injury were successfully treated with cell sheets composed of corneal epithelial cell or autologous oral mucosal cells [44, 55, 56]. As a therapy for regenerating esophageal mucosa after esophageal endoscopic submucosal dissection for resecting superficial esophageal neoplasm, applying cell sheets fabricated from autologous oral mucosal cells have been successfully conducted, preventing postoperative inflammation and stenosis [6, 45, 57]. For patients with dilated cardiomyopathy, multi-layered myoblast sheets that were fabricated from autologous skeletal myoblasts have been transplanted onto the surface of the dilated heart, resulting in dramatic improvement of the cardiac function [49]. Transplantation of layered cell sheets composed of patients’ periodontal ligament-derived mesenchymal stem cells facilitated the periodontal regeneration such as bone regeneration, cementum formation, and well-oriented collagen fibers, resulting in the improvement of the periodontal disease [58]. More recently, patients with middle ear cholesteatoma or adhesive otitis media were successfully treated with a novel method combining tympanoplasty and autologous nasal mucosal epithelial cell sheet transplantation [59].

## Cell Sheet Technology for Liver Tissue Engineering

To overcome the poor engraftment of donor hepatocytes in the setting of hepatocyte transplantation where isolated cells are directly infused into portal circulation, our team and others have investigated the possibility of “liver tissue engineering,” where functional hepatic tissues are created at extrahepatic sites in vivo. Specifically, we first engineered the hepatic tissue construct under the kidney capsules of mice by injecting primary hepatocytes mixed with Engelbreth-Holm-Swarm (EHS) gel. A higher engraftment rate of transplanted hepatocytes can be achieved by this method compared with conventional cell injection into the portal vein when the same numbers of hepatocytes are transplanted [60–62]. The engineered hepatic tissue demonstrated the same degree of liver-specific function, including the responsiveness to regenerative stimuli, protein secretion, and drug-metabolizing activity, as naïve liver [63, 64]. It is noteworthy that both morphology and functionality of the engineered liver tissues were stably maintained for over 450 days in mice [65].

We then focused on the subcutaneous site for engineering ectopic hepatic tissues because of its accessibility requiring a less invasive procedure. It has been known that primary hepatocytes transplanted into the subcutaneous space as a cell suspension do not engraft and survive over a long period of time. This is attributed to the high oxygen demand that hepatocytes require for survival [66, 67], and the blood supply at subcutaneous space being relatively poor for the maintenance of transplanted hepatocytes. To challenge these issues, we have developed a device releasing basic fibroblast growth factor (bFGF), aiming at prevascularization of the subcutaneous sites of recipient mice before hepatocyte transplantation [21, 68]. Specifically, we prepared polyethylene terephthalate mesh bag devices that are coated with polyvinyl alcohol hydrogel, filled agarose hydrogel containing bFGF in them, then implanted the devices at the subcutaneous site of recipient mice. The gradual release of bFGF stimulates angiogenesis around the device, resulting in the establishment of a local vascular network. Efficient engraftment and long-term survival of donor hepaotcytes can be achieved by transplanting to the sites after the removal of the bFGF device [21, 68].

As the next step for the development of liver tissue engineering, our group attempted to engineer cell sheets composed of primary hepatocytes in vitro using temperature-responsive culture dishes as mentioned above, and transplant the engineered cell sheets into the prevascularized subcutaneous space [21]. Primary mouse hepatocytes attached and extended on PIPAAm-grafted culture dishes. By seeding the hepatocytes on the dishes at a density of 8–10 × 10^5^ cells/cm^2^, cell confluency was efficiently achieved within 3 days. After achieving cell confluency, the cultured hepatocytes spontaneously detach from the culture dishes simply by lowering the culture temperature to 20 °C and waiting for approximately 15 min, and can be harvested as a monolayer sheet structure [21]. In contrast to the conventional cell harvesting method by enzymatic digestion, the cell-harvesting procedure using temperature-responsive culture surfaces does not damage intercellular connections. Therefore, the hepatocyte sheets maintain many types of intercellular microstructures such as desmosomes, gap junctions, and bile canaliculi, all of which are critical for preserving hepatocyte-specific functions [21]. By transplanting these engineered hepatocytes sheets at the site of prevascularized subcutaneous space, the donor primary hepatocytes demonstrated more efficient cell engraftment compared with hepatocytes transplanted as single cell suspension [21]. The transplanted sheets locally formed stratified liver structures made of recipient endothelial cells and donor hepatocyte sheet. These engineered liver constructs stably and functionally persisted for a long term (>200 days). It is noteworthy that the 3D liver system was successfully created by stacking multiple hepatocyte sheets onto each other at the same prevascularized space. Furthermore, the created 3D liver system demonstrated significant higher hepatocyte-specific function, in the stacked cell sheet number-dependent manner.

Primary hepatocytes show substantially limited cell proliferation potential in vitro. Therefore, it generally takes several days to achieve confluency, and requires certain skills to handle the engineered hepatocyte sheets because of their fragility. To prepare easy-to-handle hepatocyte sheets, we, and others, have developed several techniques. Sakai et al. utilized skin fibroblast as a feeder layer on a temperature-responsive culture dish, and cultured the hepatic cells on the fibroblast [69]. Multi-layered easy-to-handle fibroblast/hepatocyte cell sheets with a thick morphology were harvested by forceful contraction of the fibroblast. Importantly, the harvested cells possessed high liver-specific function. Furthermore, the engineered hepatocyte/fibroblast sheets expressed high levels of vascularization-associated growth factors in vitro. After the engineered sheets were transplanted to the subcutaneous space of mice, the construct developed into vascularized subcutaneous liver tissues with higher hepatic function than tissues from hepatocyte-only sheets [70].

To fabricate highly functional 3D hepatic tissues in vitro, our group has improved the cell sheet stratification technology. We succeeded in constructing liver tissues, in which the hepatocytes maintain their specific cell polarity, by sandwiching a hepatocyte sheet between two endothelial cell sheets in vitro, resulting in triple-layered hepatic tissues [71, 72•]. The construct closely mimics the in vivo liver environment because both the cell–cell and the cell–matrix interactions as observed in the native liver were reproduced. These triple-layered liver structures retained higher hepatic functionality, and could be valuable not only for drug screening tests, but as a new platform for liver tissue engineering and bioartificial liver devices.

In terms of therapeutic potential of the hepatic cell sheet as a functional tissue construct for metabolic liver support, several researchers reported their efficacy using various types of liver injury models. Rats with radiation-induced liver damage combined with partial hepatectomy were treated with subcutaneous transplantation of multilayered hepatocyte sheets fabricated with dermal fibroblast [73]. Transplanted sheets survived with additional proliferative activity, thereby maintaining the liver function, providing metabolic support for host rat liver, and improving the cell survival. Itaba et al. demonstrated the therapeutic efficacy of orthotopic transplantation of hepatic cell sheets engineered from differentiated human mesenchymal stems on acute liver injury of mice [74]. Transplantation of the cell sheets enhanced liver regeneration and suppressed liver injury, resulting in significantly improved cell survival rate. More recently, therapeutic potential of cell sheets fabricated from hepatocyte-like cells derived from induced pluripotent stem cells (iPS-HLC) for liver injury [75]. Transplantation of iPS-HLC sheets onto the liver surfaces of mice with carbon tetrachloride-induced acute liver injury successfully ameliorated the lethal liver injury. Although most of the present results are from researches using rodent models, transplantation of cell sheets composed of hepatocytes or hepatocyte-like cells seems to be feasible as a novel therapeutic modality toward liver diseases, and attempts using large animal models and subsequent clinical trials are warranted.

## Cell Sheet Technology as a Novel Therapeutic for Hemophilia

Cell-based therapy using hepatocytes are highly expected as a curative measure for hemophilia, because both FVIII and FIX are produced in the liver [23–26] and only 1–5% of increase in plasma FVIII (or FIX) activity levels can dramatically improve the bleeding symptom of hemophilia patients [27]. Clinical hepatocyte transplantation was successfully performed on the patients with congenital factor VII deficiency [3]; however, there have been no reports about their effect on hemophilia. Therefore, as a preclinical study, our group investigated the therapeutic effect of hepatocyte-based therapy on hemophilia mice. Specifically, primary hepatocytes isolated from the liver of wild-type mice were intraportally transplanted to the liver of hemophilia B mice [76], or transplanted under the kidney capsule of hemophilia B mice as a mixture with EHS gel [77, 78]. In both experiments, 1 to 3% increase of plasma FIX activity levels was observed in the recipient mice after the procedures, and persisted for a long-term period without development of FIX-neutralizing antibody. Engraftment of transplanted hepatocytes and FIX mRNA expression were confirmed by histological analyses and PCR, respectively. For further enhancing the therapeutic efficacy of hepatocyte transplantation on hemophilia B, another group utilized hepatocytes, which were genetically modified with gain-of-function FIX variant, and observed its effects [79, 80]. There is no doubt that the cell type responsible for FIX production in the liver is hepatocytes [23], whereas it has been highly controversial whether FVIII is produced by hepatocytes or liver sinusoidal endothelial cells (LSECs) [81–86]. Our group demonstrated that transplantation of wild-type hepatocytes under the kidney capsule of hemophilia A mice leads to FVIII increase in plasma [64], whereas the other groups have reported similar therapeutic effects using LSECs [82, 83]. It would be reasonable to speculate that both cell types might have FVIII expression potential, and a direct interaction between the two cell types enhances the FVIII production.

One of the major hurdles for establishing clinical hepatocyte transplantation is the poor availability of functional hepatocytes. The number of donor livers remains severely limited. And, even if they are available, the quality of these livers is frequently marginal. Furthermore, primary hepatocytes do not proliferate extensively under culture condition, and current cryopreservation technology hardly maintains primary hepatocytes in the same status as freshly isolated cells. Therefore, methods to circumvent these problems have to be explored. For that aim, three strategies have been proposed to overcome the obstacles, that is, (1) establishment of a method for propagating and preserving functional mature hepatocytes, (2) development of hepatocyte-like cells differentiated from various types of stem cells, and (3) application of genetically modified autologous cells.

### Establishment of a Method for Propagating and Preserving Functional Mature Hepatocytes

We have investigated a possible method that utilizes immunodeficient mice expressing urokinase plasminogen activator-transgenic mice under albumin enhancer/promoter, called uPA/SCID mice [87], as a host for hepatocytes. By transplanting human hepatocytes to the liver of uPA/SCID mice via spleen, the human hepatocytes integrate in the parenchyma and progressively repopulate the host mouse liver. Mice with successfully proliferated human hepatocytes can be infected with HBV and HCV. Propagated human hepatocytes also retain their synthesis ability for coagulation factors [26, 88]. In the experiments in which normal mouse hepatocytes are transplanted to uPA/SCID mice, we identified that the propagated and then isolated hepatocytes can be propagated again in the uPA/SCID mice liver. Furthermore, we have succeeded in fabricating cell sheets composed of the hepatocytes propagated in the uPA/SCID liver [89]. Although there are certainly concerns about an ethical issue and a contamination of small amount of mice-derived components, the proposed method utilizing uPA/SCID mice is advantageous in terms of enabling researchers to keep stocks of the hepatocytes and provide the fresh cells any time on demand.

### Development of Hepatocyte-Like Cells Differentiated from Various Types of Stem Cells

Stem cells, including embryonic stem cells (ES cells) or induced pluripotent stem cells (iPS cells), have recently gathered great attention in the field of regenerative medicine because of their self-replication and multipotency characteristics. Establishment of a protocol, which enables these cells to differentiate to spontaneously acquire ability for the production of coagulation factors, will secure potential cell sources for the cell-based therapy for hemophilia. We have already reported an efficient differentiation protocol for differentiating ES cells to produce functional FVIII protein [90], and a potential therapeutic effect of cell transplantation using the differentiated cells on hemophilia A mice [91]. We also conducted a comprehensive expression analysis of the coagulation-related factors in undifferentiated and differentiated iPS cells [92]. Other groups have also reported on the therapeutic effects of differentiated iPS cells transplanted to hemophilia mice [93, 94]. In these reports, hemophilia A mice were treated using iPS cells that were differentiated to endothelial/endothelial progenitor cells [94], and therapeutic levels of plasma FIX activity were successfully achieved in hemophilia B mice by injecting iPS cells that were differentiated into hepatocyte-like cells [93]. Preclinical trials using large animal models such as hemophilia dogs are expected to be performed for the translation of these excellent research results to the clinic for hemophilia patients.

### Application of Genetically Modified Autologous Cells

Cell therapy using autologous cells is more attractive than that with allogenic cells in terms of no concern of immune rejection. Engineering genetically modified autologous cells with an ability to produce functional FVIII (or FIX) will facilitate this type of therapy, called cell-based gene therapy, for hemophilia. So far, two clinical trials of cell-based gene therapy have been conducted for hemophilia patients. The first trial was performed using autologous skin fibroblasts that were transduced with FIX gene by retrovirus vector in two hemophilia B patients [95]. The peak factor IX levels in plasma were reached at 4%, but the expression was only transient. In the second trial, published by Roth et al. [96], six hemophilia A patients were implanted with genetically modified autologous skin fibroblasts. Plasmids expressing factor VIII cDNA were transduced to the fibroblasts ex vivo by electroporation. The modified autologous cells were then returned to the greater omentum of the patients using a laparoscope. This resulted in transient low-level expression of factor VIII for several months. In an attempt to build off these results using hepatocytes, we transduced human FIX gene in vitro to the primary hepatocytes isolated from hemophilia B mice, and transplanted the cells under the kidney capsule of hemophilia B mice. Engraftment of human FIX-expressing cells has been confirmed by immunohistochemistry, and significant levels of human FIX antigen were detected in the plasma (unpublished data). We also succeeded in propagating hemophilia B hepatocytes in the uPA/SCID mice livers, and transducing FIX gene to the propagated FIX-lacking hepatocytes in vivo by intravenous injection of virus vector to the mice (unpublished data). In a pilot attempt to apply cell sheet technology for the cell-based gene therapy for hemophilia, we first focused on utilizing blood outgrowth endothelial cells (BOECs).

BOECs are a subtype of endothelial progenitor cells with high proliferative capacity in vitro, and can be established from peripheral blood [97]. Initially, hemophilia A mouse-derived BOECs, lentivirally transduced with canine *FVIII* gene, were prepared and transplanted to the subcutaneous space of hemophilia A mice as a cell suspension in EHS gel [98]. Although significant levels of plasma FVIII levels were obtained after the procedure, the increased FVIII levels gradually diminished as the deterioration of the engrafted BOECs. In marked contrast, subcutaneous transplantation of the gene-modified BOECs as a cell sheet format enabled successful long-term (>300 days) engraftment of the cells with 3–5-fold higher secretion of FVIII in plasma compared with the EHS gel method [99••]. The bleeding time of the recipient mice was also significantly shortened compared with untreated hemophilia A mice. For the hemophilia B application, we are focusing on using adipose tissue-derived stem/stromal cells (ADSCs) because of their proliferation potential and accessibility [100]. Of note, we confirmed that ADSCs possess several machineries for post-translational modification of FIX protein. ADSCs were established from the subcutaneous adipose tissues of hemophilia B mice, and transduced with human FIX gene by lentivirus vector in vitro. The cells produced human FIX protein with functional clotting activity. Also, these FIX-producing ADSCs can be easily harvested as a cell sheet format. Taken together, all these results clearly suggest the feasibility of a strategy combining cell sheet engineering and gene transfer techniques as a next-generation cell-based gene therapy for liver diseases including hemophilia A and B.

## Conclusions

Cell-based therapy approaches have recently shifted from the style of suspended cell injections to the implantation of cells as an engineered tissue construct for enhancing cellular survival and prolonging the functionality of transplanted cells. Recent developments of cell sheet technology as one of the most feasible tissue engineering approaches were reviewed herein, mainly focusing on what cell sheet technology is, how to engineer liver cell sheets in vitro, techniques for achieving functional engraftment of the constructed sheets, and the evidence of practical therapeutic effects of the cell sheet transplantation for liver diseases, especially for hemophilia. Current therapy for hemophilia, in which periodic intravenous infusion of clotting factor concentrates are required throughout the whole course of life, results in tremendous economic burden worldwide. Tissue engineering-based cell therapy using allogenic cells or genetically modified autologous cells will provide stable and persistent levels of the lacked factor activity in plasma, resulting in the decrease of required factor concentrates for preventing spontaneous bleeding. Although several hurdles including cell sources, cell cryopreservation, and appropriate measure for graft rejection have to be overcome or optimized to further develop and realize the cell sheet therapy for liver diseases, the field of tissue engineering including cell sheet technology has a great potential for creating new therapeutic options for patients with various types of liver diseases.
